# Probiotics and mastitis: evidence-based marketing?

**DOI:** 10.1186/s13006-016-0078-5

**Published:** 2016-07-21

**Authors:** Lisa H. Amir, Laura Griffin, Meabh Cullinane, Suzanne M. Garland

**Affiliations:** Judith Lumley Centre, La Trobe University, Melbourne, VIC Australia; La Trobe Law School, La Trobe University, Bundoora, VIC Australia; Department of Microbiology and Infectious Diseases, Western Pacific Regional HPV Reference Laboratory, Royal Women’s Hospital, Parkville, VIC Australia; Department of Infectious Diseases and Microbiology, Murdoch Childrens Research Institute, Melbourne, Australia; Department of Obstetrics and Gynaecology, University of Melbourne, Parkville, VIC Australia

**Keywords:** Probiotics, Mastitis, Treatment, Microbiome

## Abstract

Probiotics are defined as live micro-organisms, which when administered in adequate amounts, confer health benefits on the host. Scientists have isolated various strains of Lactobacilli from human milk (such as *Lactobacillus fermentum* and *Lactobacillus salivarius*), and the presence of these organisms is thought to be protective against breast infections, or mastitis.

Trials of probiotics for treating mastitis in dairy cows have had mixed results: some successful and others unsuccessful. To date, only one trial of probiotics to *treat* mastitis in women and one trial to prevent mastitis have been published. Although trials of probiotics to *prevent* mastitis in breastfeeding women are still in progress, health professionals in Australia are receiving marketing of these products.

High quality randomised controlled trials are needed to assess the effectiveness of probiotics for the prevention and/or treatment of mastitis.

## Background

### Human microbiome and probiotics

Over 100 trillion microbes live within our bodies (tenfold greater than there are host cells), and they are vital in maintaining our health [1]. The recent surge in research about the human microbiome has demonstrated the huge variability of organisms living on and within the human body. Concerns about the way that the human microbiome may be negatively affected by modern life – the direct relationship between courses of antibiotics in childhood and the risk of inflammatory bowel disease, for example [2] – have led to investigations into the role of “good bacteria” or probiotics to reduce our vulnerability to pathogenic organisms [3].

In the past, human milk was considered a sterile fluid, and any organisms identified in milk were considered skin contaminants [4]. Now we understand that mothers’ milk contains a vast array of organisms, although the roles of these organisms have yet to be clarified.

Probiotics are defined as live micro-organisms, which when administered in adequate amounts, confer health benefits on the host [5]. A pertinent example is the use of probiotics in very low birth weight preterm infants to reduce morbidity. In a large randomised blinded controlled Australian study using a probiotic mixture (*Bifidobacterium longum* subsp. infantis BB-02, *Streptococcus thermophilus* TH-4 and *B. animalis* subsp. lactis BB-12) fed orally until term corrected age or discharge from hospital, to infants <32 weeks gestation and <1500 gm birth weight, an absolute reduction in necrotising enterocolitis (NEC) of 54 % was shown [6, 7]. This has changed practice in neonatal nurseries as a cheap and safe intervention [7]. Yet in a very similar, large randomised controlled trial in premature babies in the United Kingdom which used a different probiotic (*Bifidobacterium breve* BBG-001), no effect was reported [8].

Since probiotic activity is not just species-specific, but strain-specific, the identity of the micro-organisms used in probiotic products needs to be confirmed using molecular techniques to ensure safety and efficacy [9]. Furthermore reports of several instances where the identify of micro-organisms isolated from probiotic products differed from the information on the product label has raised concerns about documented health benefits and safety of these products [9].

Lactobacillus organisms have been shown to reduce serious infections in extremely preterm infants [10], and can reduce the risk of childhood eczema [11].

Bacteria in human milk considered to be beneficial for health – the “good bacteria” – may play a role in reducing the risk of maternal breast infection (mastitis), as well as providing the infant with a source of healthy microbes along with their nutrition. From among the several hundred species in human milk [4, 12], scientists have isolated particular species of Lactobacillus from human milk to study their effect on mothers and babies [13–16]. The effects of these Lactobacilli are strain-specific: for example, *Lactobacillus fermentum* CECT5716 has an immunostimulatory effect, in contrast to the antiflammatory effects of *Lactobacillus salivarius* CECT5713 [17].

Bacteria from women’s gastrointestinal tract are thought to travel to the mammary gland via an endogenous route, the enteromammary pathway, via dendritic cells penetrating the gut epithelium and picking up organisms, then spreading via the mucosal associated lymphoid system to the lactating mammary glands [18, 19]. Mothers who ingest *Lactobacillus* spp supplements have had the same organisms isolated from their milk and from their infants’ faeces [14, 15, 20]. Even if maternal ingestion only occurred during pregnancy, infants have been shown to be colonised by the same strain in their faecal samples up to 24 months of age [21].

### Probiotics and mastitis

Trials of probiotics for treating mastitis in dairy cows have had mixed results: some successful [22] and others unsuccessful [23]. At the local level, an intramammary infusion of *Lactobacillus lactis*, a food-grade bacterium, led to a large up-regulation of cytokines and chemokines (“a massive immune response”) [24] and neutrophils [25] capable of eliminating mastitis pathogens [24, 26].

In breastfeeding women, early mastitis can be managed by improving milk drainage from the breast, massage and applying cold packs to reduce the swelling [27]. When women are acutely ill with fever, and the red, painful, swollen area of the breast persists for more than 24 h, antibiotics are usually recommended [27, 28]. The organism most commonly isolated in infective mastitis is *Staphylococcus aureus* (*S. aureus*), and therefore anti-staphylococcal antibiotics are the mainstay of treatment (e.g., flucloxacillin) [29]. About 20 % of breastfeeding women experience at least one episode of mastitis, and 3 % of these women develop a breast abscess [30]. This is a painful collection of pus within the breast that requires drainage, usually via aspiration under ultrasound guidance [31].

Families and their health professionals are wary about using medicines during lactation [32], and even though an antibiotic such as flucloxacillin is compatible with breastfeeding [33], an alternative treatment would be welcome.

One trial in Spain set out to compare *Lactobacillus* spp probiotics with antibiotics for women with mastitis [15]. The published paper describes the results at day 21, and concludes that women receiving *Lactobacillus fermentum* or *Lactobacillus salivarius* had less pain and risk of recurrence of mastitis than women in the antibiotic arm of the trial [15]. However, the trial registry indicates that data were collected on days 7, 14 and 28 – not day 21 [34]; yet only data from one follow-up time-point (‘day 21’) are reported in the published paper [15]. Anyone suffering with mastitis would expect improvement by day 7, not three weeks later – so why was this information omitted? Furthermore, although the paper describes the trial as a blinded randomised controlled trial, the registry information explains that allocation was “non-randomised’ and women’s own doctors prescribed the antibiotics; thus the women and the clinicians were not blinded to the treatment arm. The women received a range of antibiotics: amoxycillin, amoxicillin-clavulanic acid, cotrimoxazole, erythromycin and cloxacillin – and many of these are well recognised as unsuitable for the treatment of mastitis, since *S. aureus* is commonly resistant to some of these antibiotics (amoxycillin, cotrimoxazole, erythromycin) [29]. Poor outcomes following inappropriate antibiotics is not surprising. At follow-up, the symptom reported is ‘breast pain’ which is only one of the indicators of mastitis (the triad includes lump and redness, as well as fever or ‘flu-like symptoms). In the trial, women who received antibiotics were more likely to report breast pain at day 21, but some of these women may have had symptoms of candida infection, a common sequelae to antibiotics in breastfeeding women [35].

Thus, there are seemingly many problems with the trial by Arroyo and colleagues [15, 36], and clinicians have been waiting since 2010 to hear about new trials with fewer methodological issues.

Considering whether probiotics could prevent episodes of mastitis, there is only emerging evidence. A recent paper reports on a trial to prevent mastitis using *Lactobacillus salivarus* PS2 – a different strain to the previous study – administered in late pregnancy to women with a past history of lactational mastitis [37]. The Clinical Trials Registry states the primary outcome for this RCT is: ‘Evidence of clinical mastitis confirmed by microbiological cultures and somatic cell counts [Time Frame: Weekly during the first 6 months after birth]’, yet the authors report data only to *three months* in this publication, and do not provide the somatic cell count data [37]. In clinical practice, mastitis is confirmed by the presence of breast and systemic signs and symptoms, and microbial quantitation is uncommonly used to confirm mastitis in breastfeeding women [27, 35]. In addition, the authors do not provide the difference in the incidence of mastitis used to calculate their sample size (either in the publication or the Clinical Trials Registry). The authors classified mastitis episodes as ‘acute mastitis’ (breast and systemic symptoms) and ‘subacute mastitis’ (breast inflammation only). The terms ‘acute’ and ‘subacute’ mastitis seem to be adopted from bovine mastitis research, and are rarely used to describe human lactational mastitis. Clinicians assessing a woman with localised breast inflammation would diagnosis this as a blocked duct rather than mastitis [38], and would rarely prescribe an antibiotic.

The authors state that milk samples were collected from women who did not report mastitis (‘healthy’ participants) between 91 and 100 days postpartum (*n* = 41 in the treatment arm; *n* = 23 in the placebo arm). Total bacterial counts were conducted on these samples. A statistically significant difference in mean bacterial counts was reported in healthy participants from the treatment arm compared to the placebo arm. This difference is reported as 0.19 log_10_ CFU/ml, which equates to 1.5 CFU/ml. Although this is significant statistically, we question the clinical significance of this finding and why this result was reported? Are the authors suggesting that *L. salivarius* PS2 inhibited the growth of other bacteria (potentially pathogenic)? If this is the case, would a difference of less than 2 CFU/ml be clinically significant?

The results of this prevention trial provide evidence of efficacy of this particular strain of *L. salivarius* – not currently on the market –for this group of women with a prior history of mastitis. Women in the probiotic group were less likely to experience acute mastitis (3/55; 6 %) than women in the placebo group (7/53; 13 %) in the first three months postpartum [37]. Information available in international trial registries indicate that three other trials are underway to assess the effectiveness of two strains of Lactobacillus in preventing mastitis in breastfeeding women [39–41].

Despite the paucity of completed trials, companies are actively marketing probiotics for both the treatment and prevention of mastitis in Australia.

A media release dated 8 September 2015 was entitled “Probiotics provide alternative to antibiotics for treatment of mastitis during lactation”. The media release was linked to a seminar for medical professionals held in Brisbane. Similar seminars have been held in Melbourne and Sydney, and all have been sponsored by Danone Nutricia. The media release claims that studies have shown that women treated with *Lactobacillus salivarius* “improved more quickly and had lower recurrence of mastitis than those treated with antibiotics”. The only reference cited is the paper by Arroyo et al. [15].

Since August 2015, a full page advertisement for the Nutricia probiotic product, “Mastitis relief”, has been appearing regularly in *Medical Observer*, a medical magazine for Australian general practitioners. The text says “Used at the first sign of mastitis, new Profutura Mastitis relief with Probiotic LSS may help relieve or reduce breast pain…” The only evidence provided is the same study cited from Spain [15].

Health professionals reading this advertisement would assume that there is strong evidence to support these health claims. In Australia, the Therapeutic Goods Administration (TGA) has strict regulations about the wording of claims about health and medical conditions. As we have demonstrated, mastitis is a common and potentially serious problem for new mothers, so it would seem that the TGA must have more evidence than is available in the published literature.

According to the TGA website, Nutricia received an exemption for this health claim.

Another company is also heavily marketing a probiotic product, Qiara (containing *Lactobacillus fermentum* CECT5716), at breastfeeding conferences and to health professionals, including maternal and child health nurses (email to LHA, August 2015).

Given the problematic nature of the Arroyo et al. trial [15], and the unfinished status of current trials, it is remarkable that these suppliers are already marketing their products as an effective treatment for mastitis. Indeed, doing so could even be considered as misleading or deceptive conduct.

The regulatory regimen governing health claims made in such advertisements is somewhat complex. In Australia, the TGA enforces strict regulations about the wording of claims about health and medical conditions in advertisements [42, 43]. However, most of these rules do not apply to advertisements directed exclusively to medical practitioners or nurses [42, 43].

What does still apply, is general consumer protection law; and the particular part of consumer protection law that prohibits misleading and deceptive conduct applies regardless of the fact that it is medical practitioners being advertised to, rather than the general public [44]. Cases involving this law’s predecessor (in the *Trade Practices Act 1974* (Cth)) confirm that advertising of medication in trade journals – such as publications aimed at doctors or pharmacists – can be misleading or deceptive if the precise wording of the advertisement does not reflect the methodology of the particular study which is cited [45]. In this situation, relying on one problematic study of probiotics as a treatment for mastitis [15] to market probiotics for use as both a treatment and a preventative oversteps the scientific evidence available to date.

Consumer protection law also provides that a prediction or representation about the future is misleading if there are no reasonable grounds for making it [46]. Of course, using broad language in advertisements – that probiotics may produce certain outcomes – is not tantamount to making guarantees as to the product’s efficacy for any particular user. But even a statement that is literally true can be misleading, including in circumstances where the whole truth could easily have been learned upon inquiry [47]. Courts have recognised that ‘in some circumstances it is necessary to take account of the steps that a person affected by conduct might reasonably be expected to take in order to determine whether the conduct is misleading or deceptive’ [48]. Given that most medical professionals would not respond to the current advertisements by obtaining and reading the cited study [15], including the citation in those advertisements may be seen as (wrongly) implying that the claims in the advertisement rest on more solid evidential ground.

More generally, courts have commented that if there is no scientific foundation for a statement, this may be sufficient proof that the statement is misleading [49]. Thus, even where all relevant statutory approvals have been granted for an advertising campaign for a particular product [50], advertisements can be misleading if their claims are not supported by scientific evidence [51]. This is so regardless of the advertised product’s likelihood of causing any harm to consumers [51]. As the Australian Consumer Law imposes strict liability, it is also irrelevant that a person engaging in misleading and deceptive conduct did so unintentionally or unknowingly [52, 53]. Thus, even while these suppliers are taking care to comply with the regulatory framework arising from the Therapeutic Goods Act, it appears that they may nevertheless be unwittingly making misleading statements, or implications, in their advertisements.

We conclude with comments from Professor Martin Blaser, the Past President of the Infectious Diseases Society of America and eminent New York microbiome researcher who said "… I'm generally skeptical about the many claims surrounding all the probiotics crowded on our grocery store shelves, pharmacies, and health-food stores. They are almost completely untested” [54] (p. 210). He went on to say: "We won't know if these products are doing any more good than placebos until we conduct blinded clinical trials…Unfortunately few rigorous trials of this nature have been carried out… But few of the well-conducted trials that have been performed show efficacy" [54] (p. 211).

## Conclusions

High quality randomised controlled trials are needed to assess the effectiveness of probiotics for the prevention and treatment of mastitis. Each strain of bacteria needs to be tested individually, as efficacy is not only species specific but strain-specific. The risk of safety concerns is low, but every probiotic needs to be assessed for safety, as well as efficacy [55]. Importantly, women may miss out on more effective treatments – and waste their money – unless each of the marketed probiotics is shown to be effective in reducing and treating mastitis.
